# Multi-Omics and miRNA Interaction Joint Analysis Highlight New Insights Into Anthocyanin Biosynthesis in Peanuts (*Arachis hypogaea* L.)

**DOI:** 10.3389/fpls.2022.818345

**Published:** 2022-02-16

**Authors:** Jiawei Li, Yucong Ma, Mengdie Hu, Yulu Zhao, Bin Liu, Chunmei Wang, Min Zhang, Liping Zhang, Xinlei Yang, Guojun Mu

**Affiliations:** State Key Laboratory of North China Crop Improvement and Regulation, North China Key Laboratory for Crop Germplasm Resources of Education Ministry, Laboratory of Hebei Provincial Crop Germplasm Resources, Hebei Agricultural University, Baoding, China

**Keywords:** peanut, anthocyanin, testa, multi-omics joint analysis, miRNA interaction, qRT-PCR

## Abstract

Peanut (*Arachis hypogaea* L.) is one of the most important economic and oil crops in the world. At present, peanut varieties with rich anthocyanin in testa are rare in the market, but the selection and breeding of varieties with the related traits has always attracted the attention of breeders. In this study, two peanut varieties with the pink and purple testa, G110 (G) and Z18-40 (Z) were used to conduct interaction joint analysis of multi-omics and miRNA-target gene. The anthocyanin content of Z18-40 was 7.49–8.62-folds higher than G110 on 30 DAF (days after flowering) and 45 DAF via Ultraviolet-visible Spectrophotometer (UV-5800, Shanghai, China). And then, a total of 14 candidate genes related with the anthocyanin biosynthesis were identified for correlation in different comparison groups (*R*^2^ ≥ 0.80), among of a novel gene *Ah21440* related with hydroxycinnamoyl transferase (HCT) biosynthesis was identified. In addition, Cyanidin 3-O-glucoside (Kuromanin, pmb0550) was the only common differentially accumulated metabolite (DAM) identified using multi-omics joint analysis in G1_vs_G2, Z1_vs_Z2, G1_vs_Z1, and G2_vs_Z2, respectively. Correlation analysis of miRNA-target genes and DEGs in the transcriptome shows that, *AhmiR2950*, *AhmiR398*, *AhmiR50*, and *AhmiR51* regulated to HCT and chalcone biosynthesis related candidate genes (*Ah21440*, *AhCHS*, *AhCHI*). Lastly, all of 14 candidate genes and 4 differentially expressed miRNAs were validated using quantitative real-time PCR (qRT-PCR), which trends were consistent with that of the former transcriptome data. The results provide important reference for in-depth research on the anthocyanin metabolism mechanism in peanut testa.

## Introduction

Peanut (*Arachis hypogaea* L., 2n = 4*x* = 40) is widely planted worldwide (Huang et al., 2019), with its planting area in China being only second to that of India, although the former’s total yield ranks first in the world. As a secondary metabolite in plants, anthocyanins are found in 72 genera from 27 families of flowering plants (Sarma et al., 1997) and they exhibit physiological health effects such as anti-aging, anti-mutation, prevention of cardiovascular diseases, liver protection and anti-cancer (Li et al., 2018). As greater concern is now being shown for health issues, anthocyanin biosynthesis has, therefore, become a highly studied compound for researchers (Wu, 2018). Testa colors are closely associated with anthocyanin content, with the different types of anthocyanin determined by the 3′,4′,5′-OH numbers in the tricyclic compound C6-C3-C6 (Yao et al., 2004). Based on these differences, blue-violet delphinidin, magenta pelargonidin, red cyanidin along with their derivatives (i.e., peonidin, petunidin, and malvidin) form the six main anthocyanins present in nature (Jaakola, 2013).

The pathway for anthocyanin biosynthesis has already been determined (Gao et al., 2020). Basically, delphinidin, pelargonidin and cyanidin are synthesized from phenylalanine with phenylalanine ammonia-lyase (PAL), 4-coumarate, coenzyme a ligase (4CL), chalcone synthase (CHS), chalcone isomerase (CHI), flavanone-3-hydroxylase (F3H), dioxanonol-4-reductase (DFR), and anthocyanidin synthase (ANS) (Song et al., 2019). The common substrates dihydrokaempferol (DHK), dihydroquercetin (DHQ), and dihydromyricetin (DHM) produce flavonols and proanthocyanidins (PAs) under the action of flavonol synthase (FLS), leucoanthocyanidin reductase (LAR), and anthocyanidin reductase (ANR) (Trainin et al., 2021). Proanthocyanins are plant natural products that are beneficial for human and livestock health (Lu et al., 2021). During the process, PAs are irreversibly converted to anthocyanin under high temperature conditions, which further deepens the colors of plants (Deng, 2018). Color changes in various plants have been reported to be related to anthocyanin biosynthesis (e.g., white clover (*Trifolium repens*) (Duan et al., 2020), alfalfa (*Medicago sativa*) (Xue et al., 2019), white primrose (*Primula vulgaris*) (Li et al., 2019), strawberry (*Fragaria × ananassa*) (Zhang et al., 2018), and other plants.

The mRNA Interfering Complementary RNA (miRNA) or antisense RNA has been demonstrated to regulate different types of genes (Stephenson and Zamecnik, 1978; Izant and Weintraub, 1984). In this respect, for the rice genome Rhoades et al. (2002) identified 34 miRNA regulatory loci that were associated with cell growth and differentiation. AtmiR156 has a positive regulatory effect on anthocyanin and upregulate the gene DFR by inducing and explaining the expression of SPL9 in Arabidopsis (Gou et al., 2011). miR828 in tomato can negatively regulate the biosynthesis of anthocyanin by acting on the target gene S1Myb7-lik (Jia et al., 2015). Therefore, in-depth studies on miRNA-target gene interactions in the regulation of peanut testa colors could be useful.

Nowadays, high-throughput omics methods, especially transcriptomics and metabolomics, have been widely used by botanists and deepen their comprehension of different biological pathways (Raza, 2020). Study investigated the differentially expressed genes (DEGs) of anthocyanin synthesis using transcriptome-metabolome joint analysis for the testa of both parents, and analyzed the miRNA-target gene interactions. The results of this study are expected to provide helpful insights into the cultivation of peanut varieties with concentrated anthocyanin. Additionally, this work is expected to be a reference for in-depth research on the metabolic mechanism of peanut testa anthocyanin.

## Materials and Methods

### Sample Preparation

In this study, the purple testa peanut variety with high oleic acid (O: 79.52%; L: 5.48%; O/L: 14.51%), Z18-40, obtained from the cross of “G110 × Zizhenzhu.” G110 with pink testa and high-oleic acid (O: 74.62%, L: 5.48%; O/L: 13.62) was used as maternal parent and Zizhenzhu with purple testa and normal oleic acid (O: 44.83%, L: 35.17%; O/L: 1.27) was used as paternal parent. Two kompetitive allele-specific PCR (KASP) markers, A004807 and A004808 were used to identify F_2_ offspring and a total of 66 high-oleic acid progenies with genotype *aabb* were detested and continuously self-pollination up to F_7_, resulting in superior lines namely Z18-40 (Supplementary Figure 1). Z18-40 was one of the new varieties both enrich in anthocyanin and high oleic acid. During the breeding process, different testa color existed between G110 and Z18-40. This phenomenon attracted us to detect the molecular mechanism of anthocyanin. G110 and Z18-40 were planted separately at Yixian station (Baoding, China) on May 2020, and were labeled with a tag after flowering between July and October. Samples consisting of 1–3 g of testa were taken on both 30 DAF and 45 DAF for G110 (G1, G2) and Z18-40 (Z1, Z2) before being wrapped in tin foil. They were then flash-frozen in liquid nitrogen for 5 s and stored at –20°C. Three biological replicates for each sample were sent to Gene Denovo Biotechnology Co. (Guangzhou, China) for sequencing.

### Measurement of Differences in the Values of Testa Color

The a, b, and L values of testa at 30 DAF, 35 DAF, 40 DAF, 45 DAF, 50 DAF, and 55 DAF for the two varieties were measured with a colorimeter (CR-10Plus, Japan) using three biological replicates. One-way ANOVA was performed using SPSS26 and the size of the difference in color (ΔE) was calculated with 30 DAF as the reference according to the following equation:

$$\Delta \,\text{E}=\sqrt[{2}]{{\left(\Delta \,L\right)}^{2}+{\left(\Delta \,a\right)}^{2}+{\left(\Delta \,b\right)}^{2}}$$

ΔE values 0.00–0.25, 0.25–4.00, and >4.00 were considered to be an ideal, an acceptable and substantial differences, respectively. Peanuts were sectioned and each section was stained with 0.5% vanillin solution for 20 min. Changes in the testa color at 30 DAF and 45 DAF were then measured by stereomicroscopy (XDL-7000, China). In this case, the background was processed using Adobe Photoshop 2021.

### Transcriptome Analysis

The testa of G110 and Z18-40 at 30 DAF and 45 DAF were collected. Testa samples were flash-frozen using liquid nitrogen and stored at -80^°^C until RNA extraction. RNA was extracted by Trizol precipitation with three biological replicates (Kingston et al., 2001). The purity and integrity of RNA were analyzed using NanoDrop ND-1000 UV/Vis spectrophotometer (Thermo Fisher Scientific, Wilmington, DE, United States) and Agilent 2100 Bioanalyzer (Agilent, United States), respectively. Specific samples (RIN ≈ 10; 28S/18S ≥ 1.5; 1.7 < OD260/OD280 < 2.0) were selected for transcriptome sequencing and 12 libraries of complementary DNA (2 varieties × 2 periods × 3 replicates) were constructed using a five-step process prior to sequencing on the Illumina platform.^1^ High-quality sequences were then compared with the tetraploid cultivar peanut reference genome^2^ using the HISAT2 system (Kim et al., 2015).^3^ The fragments per kilobase per million (FPKM) values of the two sets of samples were also recorded while the ratio of FPKM (Fold Change, FC) as well as the false discovery rate (FDR) for comparing differential expression were calculated using DESeq2 (Love et al., 2014), with thresholds of | log2FC| ≥ 1 and FDR < 0.05 being applied in order to screen for the differentially expressed genes (DEGs).

### Metabolome Analysis

To evaluate the testa anthocyanin content of G110 and Z18-40, the method was used for content measurement and improvement as Wrolstad et al. (2001). Firstly, testa samples at 30 DAF and 45 DAF, frozen immediately in liquid nitrogen and stored at -70°C until use. Total anthocyanin content was measured using a spectrophotometric (UV-5800, Shanghai, China) method. In short, total anthocyanin was extracted using 1% HCl/methanol in the dark at 4°C overnight with occasional shaking. The extracts were centrifuged at 10,000 r for 10 min, and the supernatant was centrifuged at 12,000 r for 15 min and measured at 530 and 657 nm for absorbance determination. The equation A530 - 0.25 × A657 was used to compensate for the absorption of chlorophyll and its degradation products at 530 nm. Total anthocyanin content was calculated using the subtracted absorbance/fresh weight. The anthocyanin analysis of each sample was repeated three times using three independent biological replicates.

Liquid chromatography tandem mass spectrometry (LC-MS/MS) was used for the qualitative and quantitative determination of the different testa. After meeting the liquid phase and mass spectrometry conditions, methanol was used as a solvent and stored at -20°C. The samples were diluted with 70% methanol to yield different gradients before mass spectrometry, and were then extracted with methanol. The reagents were chromatographically pure ethanol and acetonitrile from Merck (United States) as well as standards (analytical purity) from BioBioPha or Sigma-Aldrich. The experiment was conducted with three biological replicates. The structural analysis of metabolites was carried out based on existing mass spectrometry public databases such as HMDB,^4^ MoToDB,^5^ and METLIN.^6^ The variable importance in the projection (VIP) > 1 and *p* < 0.05 was the threshold in order to screen DAMs.

### Interaction of miRNAs-Target Gene

After Illumina sequencing, discard any low-quality reads, adaptors, contaminated sequences, and sequences shorter than 18 nt. Only the remaining high-quality sequences between 18 and 30 nt were further analyzed. All unique sequences were compared with the GeneBank tabase^7^ to identify miRNAs. Known miRNAs were identified using a BLAST search against the miRNA database miRBase release 20.^8^ Reads that were not annotated for any category were used to predict novel miRNAs using the miRNA prediction program MIREAP.^9^ Calculate the expression amount of miRNA in each sample, and use the TPM (Transcripts Per Million) algorithm to normalize the expression amount. Differential expression analysis of miRNAs was performed using edgeR software (Robinson et al., 2010), with | log2FC| ≥ 1 and FDR < 0.05 selected as screening criteria to identify DEGs. Prediction and enrichment analysis of differentially expressed target genes by clusterProfiler among sample groups were analyzed, additionally, the enrichment degree of the pathways were analyzed by enrichment factor, and significance of enrichment was calculated by Fisher’s exact test.

### Quantitative Real-Time PCR Analysis

#### Analysis of Differentially Expressed Genes by Quantitative Real-Time PCR

Quantitative real-time PCR (qRT-PCR) was applied to verify the transcription levels of candidate genes. The same RNA samples in high-throughput sequencing were used for qRT-PCR. Each 10-μl qRT-PCR reaction mixture contained 1 μl of 10-fold diluted first-strand complementary DNA, 0.3 μl of each primer (10 μM), and 5-μl 2 × PowerUP™ SYBR™ Green Master Mix (Applied Biosystems, Carlsbad, CA, United States). A Bio-Rad CFX96 real-time PCR system was used under the following conditions: 50°C for 2 min, 95°C for 2 min, followed by 40 cycles of a denaturing step at 95°C for 10 s, an annealing step at 60°C for 20 s, and an extension step at 72°C for 45 s. For this purpose, primers were designed using Premier 5.0 software and the experiment was carried out with three biological as well as three technical replicates (Supplementary Table 1). The *ACT7* was used as an internal reference gene, to analyze the qRT-PCR results by 2^–ΔΔCt^ analysis (Livak and Schmittgen, 2001). *T*-tests were used to compare differences in gene expression.

#### Analysis of miRNAs by Quantitative Real-Time PCR

Quantitative real-time PCR was applied to verify the sequencing levels of candidate miRNAs. The same RNA samples in high-throughput sequencing were used for qRT-PCR. Firstly, the reverse transcription system was configured according to Tiangen kit instructions (miRcute Plus miRNA First-Strand cDNA Synthesis, Beijing, China) for synthesizing the first strand. The 20-μl reverse transcription system contained 10-μl 2 × miRNA RT Reaction Buffe, 2-μl miRNA RT Enzyme Mix, 1-μl Total RNA and 7-μl RNase-Free ddH_2_O. The reverse transcription program was used under the following conditions: 42°C for 60 min and 95°C for 2 min. The reverse primer used universal primer and the design and improvement of the forward primer followed the methods such as Chen et al. (2005) by using SnapGene 4.1.8. The 5 s rRNAs was used as an internal reference (Supplementary Table 2). Each 20-μl qRT-PCR reaction mixture contained 10-μl 2 × miRcute Plus miRNA and Premix (with SYBR&ROX), 0.3-μl Forward Primer (10 μM) and Reverse Primer (10 μM), 1-μl the first strand cDNA of miRNA and ddH_2_O Until 20-μl. The detection was carried out according to the instructions of the fluorescence quantification kit (miRcute Plus miRNA qPCR Detection Kit, Beijing, China). The reaction program of qRT-PCR was designed under the following conditions: 40 cycles of qRT-PCR used a two step reaction which consisted of a pre-denaturation step at 95°C for 15 min, PCR cycle step at 94°C for 20 s, and an annealing and extension step at 60°C for 34 s. The analysis method of qRT-PCR results was the same as the above methods.

## Results

### Analysis of Testa Color Differences

**FIGURE 1:**
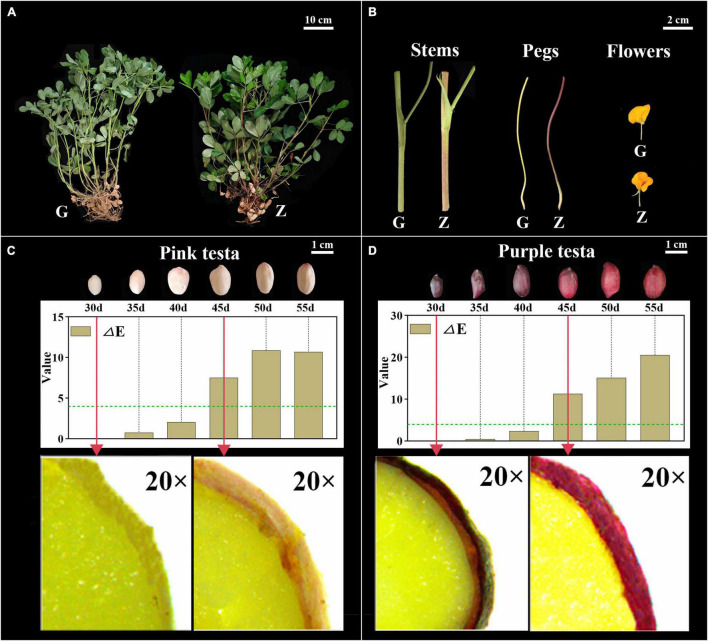
Observation and Identification of botany phenotypes between G110 and Z18-40. Panel **(A)** shows the color difference comparison of the whole plants between G110 (G) and Z18-40 (Z). G110 plant is on the left, Z18-40 plant is on the right, and the scale bar is 10 cm. Panel **(B)** shows the color difference comparison of the stems, pegs and flowers between G and Z, and the scale bar is 2 cm. Panels **(C,D)** show the testa color changing trend between G and Z in six growth periods, and the scale bar is 1 cm. In addition, “ΔE” value of G and Z testa is determined in six periods and the green dotted line means ΔE = 4. The red arrows point to the testa of 30 DAF and 45 DAF in G and Z, respectively, which is observed with a stereo microscope on 20-folds magnification.

The color of Z18-40 plants was obviously darker than that of G110 plants (Figure 1A). The stems and pegs of G110 were of green color, while those of Z18-40 were purple-red. In the case of flowers, those of G110 were faint yellow in color, while those of Z18-40 were orange-red, with the red color being prominent in the flag and wing petals (Figure 1B). In terms of testa color, a visual change from light to dark was observed during all six periods. This was particularly obvious from microscopic observations which showed that G110 was pinkish white at 30 DAF before gradually changing to pinkish red at 45 DAF. Similarly, Z18-40 was purple-black at 30 DAF and turned purple-red at 45 DAF. The results of color measurements showed that the “a” values of G110 were 11.2–15.2 (*p* < 0.05), the “b” values were 12.1–16.6 (*p* < 0.05) and the “L” values were 31.2–41.7 (*p* < 0.05). The ΔE value changed from 2.34 to 10.89 during the whole pod growth period, and ΔE > 4 after 45 DAF. The “a” values of Z18-40 were 2.1–7.8 (*p* < 0.05), “b” values were 2.2–5.9 (*p* < 0.05) and “L” values were 0.1–6.7 (*p* < 0.05). The ΔE value changed from 0.47 to 20.54 during the whole pod growth period, and ΔE > 4 after 45 DAF (Figures 1C,D).

### Transcriptome Analysis of Anthocyanin Between Two Peanut Varieties

**FIGURE 2:**
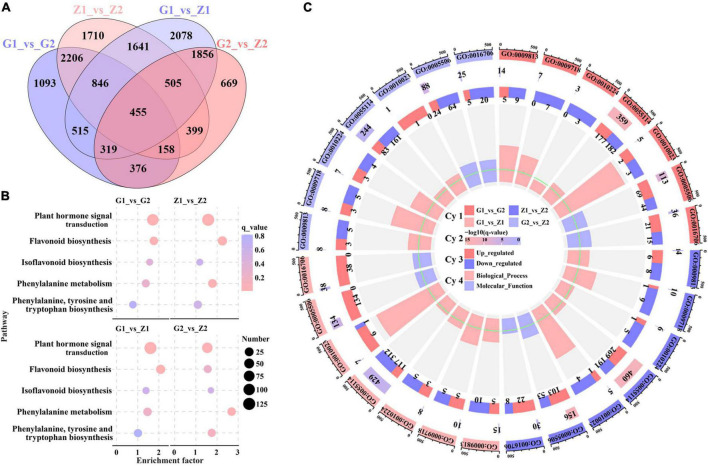
Screening and enrichment analysis of anthocyanin DEGs. Venn diagrams **(A)** displaying overlap between DEGs identified in peanut testa in G1_vs_G2, Z1_vs_Z2, G1_vs_Z1 and G2_vs_Z2. The number on the part without overlapping colors represents the DEGs in a single comparison group. The number on the overlapping color part indicates the DEGs shared among different comparison groups. Picture **(B)** shows KEGG pathway enrichment analysis among the above four comparison groups, in which the abscissa means the enrichment factor, the ordinate means the enrichment pathways, the size of the circles means the number of DEGs, and the color of each circle means q_value. Picture **(C)** is made up of four cycles. Cycle 1 (Cy1, the outermost cycle) indicates 7 GO Classify2 localized to the four comparison groups respectively. Cycle 2 (Cy2, the secondary outer cycle) means the number of DEGs corresponding to the GO Classify2. The color of the rectangles below the number indicates the q value (-log10). Cycle 3 (Cy3, the sub-internal cycle) means the number of upregulated and downregulated genes corresponding to GO Classify2. Cycle 4 (Cy4, the innermost cycle) means the enrichment scope of GO Classify2, which include biological process and molecular function. The green circular line means the enrichment factor = 1.

To further explore the molecular mechanism of peanut testa pigment differences, 12 cDNA libraries were constructed for the testa at 30 DAF and 45 DAF. This generated 79.87 Gb of clean data, of which 5.91 Gb was obtained for each sample (92.4% of reads ≥ Q30). Sequence comparison between the reads of each variety and the reference genome indicated a similarity level of 84.1–96.1%. Furthermore, a total of 26,840 DEGs were identified in the G1_vs_G2, Z1_vs_Z2, G1_vs_Z1 and G2_vs_Z2 comparison groups, with 3,387, 4,643, 3,111, and 1,883 upregulated genes and 2,581, 3,277, 5,104, and 2,854 downregulated genes, respectively. These included the anthocyanin biosynthesis-related genes *AhPAL*, *AhCHS*, *AhCHI*, *Ah4CL*, *AhF3H*, *AhFLS*, *AhDFR*, *AhANR*, *AhLAR*, *AhLDOX* as well as the regulatory genes *AhbHLH* and *AhbZIP* genes (Supplementary Figure 2). Venn analysis further revealed that 455, 1,824, and 6,994 genes overlapped when during four-, three-, and two-group comparisons, respectively, while 1,093, 1,710, 2078, and 669 genes were unique to the above comparison groups (Figure 2A). In addition, results of KEGG analysis indicated that 119, 125, 124, and 122 pathways were enriched in the four comparison groups, and plant hormone signal transduction (ko04075), flavonoid biosynthesis (ko00941), isoflavonoid biosynthesis (ko00943), phenylalanine metabolism (ko00360) and phenylalanine, tyrosine and tryptophan biosynthesis (ko00400) were related to anthocyanin biosynthesis. The results of four comparisons indicated that there were 100, 66,123, and 126 DEGs enriched in plant hormone signal transduction and 20, 13, 34, and 32 DEGs enriched by flavonoid biosynthesis, which were the two key differential enrichment pathways in anthocyanin biosynthesis (Figure 2B and Supplementary Table 3). GO enrichment analysis resulted in two GO Classify1 (Biological process and Molecular function) and seven GO Classify2 [flavonoid biosynthetic process (GO:0009813), anthocyanin-containing compound biosynthetic process (GO:0009718), response to UV-B (GO:0010224), oxidation-reduction process (GO:0055114), proanthocyanidin biosynthetic process (GO:0010023), Iron ion binding (GO:0005506), and oxidoreductase activity (GO:0016706)] related to anthocyanin biosynthesis. oxidation-reduction processes were enriched with 359, 460, 429, and 244 DEGs for the four comparison groups. The enrichment factors for the proanthocyanidin biosynthetic process in the four groups (except for G2_vs_Z2) were 5.98, 4.41, and 5.75, which was the highest in GO Classify2 (Figure 2C and Supplementary Table 4).

### Metabolome Analysis of Anthocyanin

**FIGURE 3:**
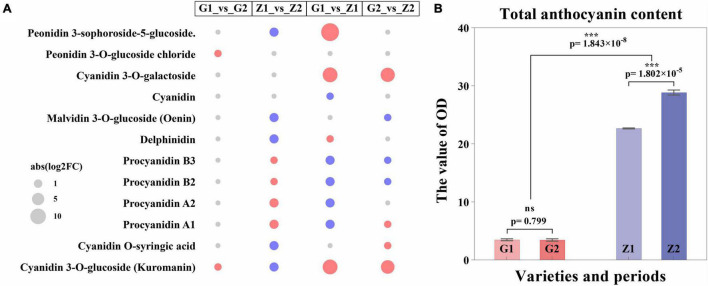
Expression regulation of DAMs and differential analysis of anthocyanin content. Panel **(A)** shows the expression regulation of DAMs, in which the abscissa means the comparison groups, the ordinate means DAMs, the size of the circle means abs (log2FC), the red means upregulated expression, and the blue means downregulated expression. Panel **(B)** shows differential analysis of anthocyanin content, where the abscissa means varieties and periods. G1 and G2 means 30 DAF and 45 DAF of G110, respectively, Z1 and Z2 means 30 DAF and 45 DAF of Z18-40, respectively. The ordinate means the OD value (OD/g). “ns” means no significant difference (*p* > 0.05) and “***” means significant difference (*p* < 0.001).

The metabolome analysis of anthocyanin resulted in the detection of 12 DAMs in a total of 16 metabolites. Two, nine, nine and seven DAMs, with log2FC values of 0.7–1.0, -1.8 to 1.6, -1.5 to 15, and -0.9 to 7.7 were identified in G1_vs_G2, Z1_vs_Z2, G1_vs_Z1, and G2_vs_Z2, respectively. The procyanidin B3, B2, A2, and A1, were all upregulated in Z1_vs_Z2 but downregulated in G1_vs_Z1. DAMs were involved in regulating expression obviously in two comparison groups as follows: (1) In G1_vs_Z1, log2FC > 5 for peonidin 3-sophoroside-5-glucoside, cyanidin 3-O-galactoside, cyanidin 3-O- glucoside, and kuromanin were significantly upregulated; (2) In G2_vs_Z2, log2FC > 5 for cyanidin 3-O-galactoside, cyanidin 3-O-glucoside, and kuromanin were significantly upregulated (Figure 3A, Supplementary Figure 3, and Supplementary Table 5).

Two pathways for anthocyanin biosynthesis (ko00942) and flavonoid biosynthesis (ko00941) were obtained based on KEGG enrichment analysis. The total anthocyanin contents at 30 DAF and 45 DAF showed that the anthocyanin content of Z18-40 was 7.49–8.62-folds higher than that of G110 (*p* = 1.843 × 10^–8^), with highly significant differences. For the same variety, the differences were not significant (*p* = 0.799) between G1 and G2, while were highly significant (*p* = 1.802 × 10^–5^) between Z1 and Z2 (Figure 3B and Supplementary Table 6).

### Transcriptome and Metabolome Joint Analyses of Anthocyanin

**FIGURE 4:**
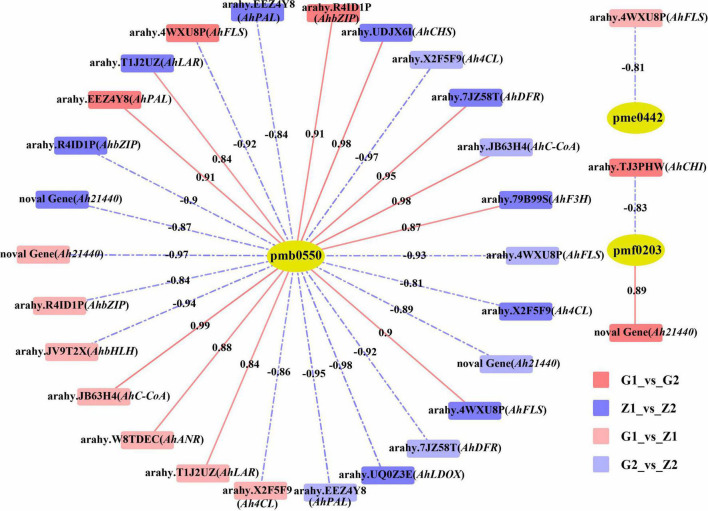
Correlation analysis between DAMs and candidate DEGs. The pmb0550, pme0442, and pmf0203 above the yellow ellipses indicate cyanidin 3-O-glucoside (Kuromanin), delphinidin and peonidin 3-O-glucoside chloride, respectively. DEGs are labeled above the rectangles. Rectangles with red, blue, light red and light blue indicate G1_vs_G2, Z1_vs_Z2, G1_vs_Z1, and G2_vs_Z2, respectively. Red solid lines and blue dashed lines indicate positive correlation and negative correlation between DEGs and DAMs, on which are the correlation value.

Based on the DEGs and DAMs of the four comparison groups obtained by transcriptome and metabolome analysis, correlation analysis was carried out in the enrichment pathway of flavonoid biosynthesis (ko00941). In. G1_vs_G2, differential gene expression was correlated with peonidin 3-O-glucoside chloride (pmf0203) as well as cyanidin 3-O-glucoside (Kuromanin, pmb0550), with 25 DEGs, 12 positive and 13 negative correlations. For the Z1_vs_Z2 comparison, differential gene expression was associated with cyanidin 3-O-glucoside (Kuromanin, pmb0550) and delphinidin (pme0442), and involved 33 DEGs, of which 15 were positively and 18 were negatively associated. In G1_vs_Z1, differential gene expression was associated with cyanidin 3-O-glucoside (Kuromanin, pmb0550), procyanidin A1 (pme0430), procyanidin B2 (pme0434), and delphinidin (pme0442). For this group, 24 DEGs were associated, with 6 being positively and 18 being negatively associated. Finally, in G2_vs_Z2, differential gene expression was associated with cyanidin 3-O-glucoside (Kuromanin, pmb0550) and was associated with 29 DEGs, with 10 showing positive and 19 showing negative associations (Supplementary Table 7). Lastly, we filtered 14 key candidate genes in different comparisons and determined their chromosomal (Chr) position, *Ah21440* (novel Gene), *AhCHS* (arahy. UDJX6I, 8633464-8632413), *AhCHI* (arahy. TJ3PHW, 152096417-152097158), *AhPAL* (arahy. EEZ4Y8, 391227-389497), *Ah4CL* (arahy. X2F5F9, 2943040-2944560), *AhC-CoA* (arahy. JB63H4, 15250522-15251181), *AhF3H* (arahy. 79B99S, 11007614-11007083), *AhFLS* (arahy. 4WXU8P, 19358597-19357631), *AhDFR* (arahy. 7JZ58T, 154671103-154670569), *AhLAR* (arahy. T1J2UZ, 110006213-110006838), *AhANR* (arahy. W8TDEC, 126128334-126128550), *AhLDOX* (arahy. UQ0Z3E, 13645491-13647176), *AhbHLH* (arahy. JV9T2X, 4730198-4731619) and *AhbZIP* (arahy. R4ID1P, 74478269-74477851), associated with anthocyanin biosynthesis (*R*^2^ ≥ 0.80) (Figure 4).

### Interaction of miRNAs and Target Genes and Screening of Key Candidate Genes

**FIGURE 5:**
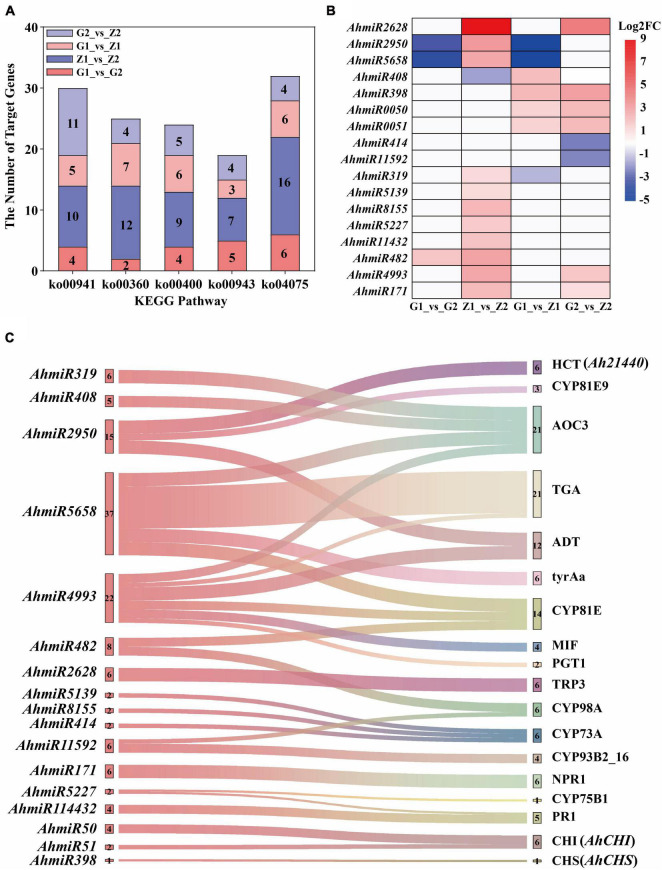
Interaction analysis of miRNAs and target genes. The five bar graphs in panel **(A)** are composed of red, blue, light red, and light blue components, representing G1_vs_G2, Z1_vs_Z2, G1_vs_Z1, and G2_vs_Z2, on which is the number of the target genes, respectively. The abscissa means the KEGG pathways, named flavonoid biosynthesis (ko00941), phenylalanine metabolism (ko00360), tryptophan biosynthesis (ko00400), isoflavonoid biosynthesis (ko00943), and plant hormone signal transduction (ko04075). The ordinate means the number of target genes. Panel **(B)** shows that the regulation of differentially expressed miRNAs in the above four comparison groups. The abscissa means the comparison groups, and the ordinate means the differentially expressed miRNAs. The red means upregulated expression, the blue means downregulated expression, and the white means that there is no differential expression. Panel **(C)** shows the interaction of miRNA-target genes. The left is the differentially expressed miRNAs and the number beside miRNAs means the number of corresponding target genes. The right is the ko_names of the target genes, and the number beside ko_names means the number of miRNAs.

A total of 131.09 Gb clean tags were obtained after miRNA sequencing of the four peanut testa samples and through comparison with the miRBase database, 28 (0.54%) existing miRNAs, 430 (9.35%) known miRNAs and 407 (0.43%) novel miRNAs were identified. A total of 531 differentially expressed miRNAs were also screened for the four comparison groups, with 20, 101, 93, and 63 upregulated and 22, 115, 59, and 58 downregulated expression levels, respectively. Moreover, 4,174, 10,047, 5,037, and 5,791 target genes were predicted for the four groups. Additionally, the results of KEGG enrichment analysis indicated that 99, 129, 110, and 126 metabolic pathways could be identified for the four comparison groups. Among these, five pathways were related to anthocyanin biosynthesis and they included 30, 25, 24, 19, and 32 differentially expressed target genes (Figure 5A). The analysis of differentially expressed target genes and miRNAs interactions further showed that 17 miRNAs were localized within the comparison groups, with 1, 11, 4, and 6 upregulated gene expression and 2, 1, 3, and 2 downregulated gene expression, respectively (Figure 5B). Correlation analysis of target genes and DEGs in the transcriptome shows that the *Ah21440* (novel Gene), *AhCHS* (arahy. UDJX6I), and *AhCHI* (arahy. TJ3PHW), which were related to anthocyanin biosynthesis, were regulated by *AhmiR2950*, *AhmiR398*, *AhmiR50*, and *AhmiR51* respectively, and were identified as the major miRNAs involved in anthocyanin regulation (Figure 5C and Supplementary Table 8).

### Verification of Candidate Genes and miRNAs by Quantitative Real-Time PCR

**FIGURE 6:**
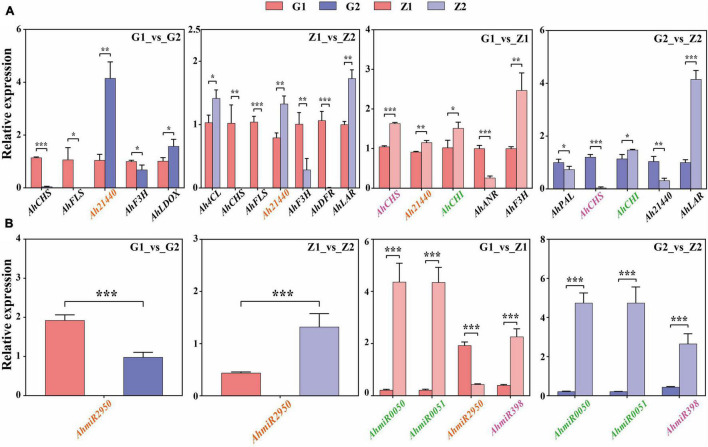
Verification of candidate DEGs and miRNAs by qRT-PCR. Panel **(A)** shows that the verification of candidate DEGs by qRT-PCR in G1_vs_G2, Z1_vs_Z2, G1_vs_Z1, and G2_vs_Z2, respectively. The abscissa means the candidate DEGs and the ordinate means the relative expression. “*” indicates *p* < 0.05, “**” indicates *p* < 0.01, and “***” indicates *p* < 0.001. Panel **(B)** shows that the verification of differentially expressed miRNAs by qRT-PCR. The abscissa indicates the candidate miRNAs, the ordinate indicates the relative expression. “*” indicates *p* < 0.05, “**” indicates *p* < 0.01, and “***” indicates *p* < 0.001. The target genes in panel **(A)** that have an interaction relationship with the miRNAs in panel **(B)** are marked with the same color.

Fourteen DEGs related to anthocyanin biosynthesis were screened through multi-omics and miRNA joint analysis results and validated by qRT-PCR. In G1_vs_G2 comparisons, nine DEGs were validated, including four upregulated genes and five downregulated genes. Two of the four upregulated genes and three of the five downregulated genes showed significant differences in expression. In Z1_vs_Z2, ten DEGs, including four upregulated and six downregulated genes, were validated. Three of the four upregulated genes and four of the six downregulated genes showed significant differences in expression. In G1_vs_Z1, nine DEGs, including four were upregulated genes and five were downregulated genes. Four upregulated genes and one of the five downregulated genes showed significant differences in expression. Finally, in G2_vs_Z2, 10 DEGs, including three upregulated genes and three in seven downregulated genes, were validated. Two of the three upregulated genes and three of the seven downregulated genes showed significant differences in expression. The key candidate genes, *Ah21440* (novel Gene), *AhCHS* (arahy. UDJX6I), *AhCHI* (arahy. TJ3PHW), *AhPAL* (arahy. EEZ4Y8), *Ah4CL* (arahy. X2F5F9), *AhF3H* (arahy. 79B99S), *AhFLS* (arahy. 4WXU8P), *AhDFR* (arahy. 7JZ58T), *AhLAR* (arahy. T1J2UZ), *AhANR* (arahy. W8TDEC), *AhLDOX* (arahy. UQ0Z3E) related to the anthocyanin biosynthesis process, were all validated and significantly different for the four comparison groups and these trends were consistent with the transcriptome data (Figure 6A and Supplementary Table 9).

Four miRNAs related to target genes interaction were screened through interaction of miRNA and target genes and validated by qRT-PCR. In G1_vs_G2 comparisons, *AhmiR2950* was downregulated. In Z1_vs_Z2, *AhmiR2950* was upregulated. In G1_vs_Z1, *AhmiR50*, *AhmiR51*, and *AhmiR398* were upregulated, and *AhmiR2950* was downregulated. In G2_vs_Z2, *AhmiR50*, *AhmiR51*, and *AhmiR398* were all upregulated. These trends were consistent with the miRNA sequencing data (Figure 6B and Supplementary Table 10).

## Discussion

### Advantages of Research Materials

Two peanut varieties with different level of anthocyanin were selected, and pink testa (3.62 OD/g) was used as the maternal parent while purple testa (25.99 OD/g) was used as the paternal parent. These characteristics were selected so that purple testa could be distinguished as a true hybrid based on testa color in F_2_ populations, thus improving the accuracy and efficiency of hybrid selection. Similar cross combinations were used in the study of Chen et al. (2021). At the same time, stable high oleic acid homozygous individual plants could be selected from F_2_ by way of KASP. This method is more practical than SSR (Zhang et al., 2014) and AS-PCR (Li, 2015). The pink testa of the female parent G110 and the superior Z18-40 purple testa of F_7_ were analyzed. Simultaneously, the testa color of the two varieties was different and were adopted to explore the molecular mode of anthocyanins in this research.

### Testa Color-Based Identification

The botany phenotypic results showed that the testa color gradually darkened with the developmental period. Purple testa showed purple-red color expressed on 45 DAF, and this color change was observed, in whole plants, stems, fruit needles, and flowers. Obviously, “L” value was inversely related to anthocyanin content (Zhang et al., 2020). The results hint that the metabolic mechanism of anthocyanin in varieties with high anthocyanin content may impact all parts of the plant. The ΔE > 4 after 45 DAF showed significant color differences, thereby suggesting that 45 DAF was the key period for testa color change. Li et al. (2017) explicitly suggested that the period of peak color as a result of gene expression occurred between 40 DAF and 45 DAF, which is consistent with our results.

### Regulation of Structural Candidate Genes and Metabolites

*PAL* is the first structural gene in anthocyanin biosynthesis, with Bar-Akiva et al. (2010) demonstrating that PAL activity can promote the formation of anthocyanins. In this study, although G 110 was higher than Z18-40 in *AhPAL* at both 30 DAF and 45 DAF, no significant differences were observed in the later developmental period, thus implying that *AhPAL* may be the first key DEG for color changes in pink testa. In a study involving pink testa peanuts, Wan et al. (2018) demonstrated that the expression of *CHS* increased as the reproductive period progressed. In this study, *AhCHS* continued to be highly expressed in G110, but was only observed for the first time in Z1 at 30DAF. It was suggested that *AhCHS* could be the first key DEG for early color differentiation in peanut testa, with these results being consistent with previous studies (Hu et al., 2021a). It was also found that the expression level of *CHI* was positively correlated with anthocyanin content (Liu et al., 2019), and that *CHI* activity could increase the flavonoid content (Li et al., 2006). Additionally, even though *AhCHI* was consistently highly expressed in Z1, it was first highly expressed in Z2. The result hints that the stage from *AhCHS* to *AhCHI* is a critical period for purple testa pigment accumulation.

*F3H* is a relay gene in anthocyanin biosynthesis, whose high expression can result in orange-red petals in dahlias (Enrique et al., 2020). In this study, *AhF3H* was highly expressed in Z1, and it led to the accumulation of the substrates of DHK, DHM, and DHQ from the *AhDFR* and *AhFLS* genes, thus forming a large amount of anthocyanin while deepening testa color. This was consistent with the results on the phenotypes of Z1. In G1_vs_G2, *AhF3H* was downregulated, and it suggested that the accumulation of substances for pink testa occurred at an early stage. Regarding *FLS* and *DFR*, they represent two competing genes which lead to the flavonol and anthocyanin pathways, respectively (Lim et al., 2016). In this study, *AhFLS* was downregulated in G1_vs_G2, while *AhDFR* was not differentially expressed. These results clearly indicated that, in the downstream pathway, *AhFLS* catalyzed large amounts of substrates within the flavonol pathway, while only few substrates were converted by *AhDFR*, hence resulting in less anthocyanin accumulation. Similarly, *AhFLS* was downregulated in Z1_vs_Z2, but in this case, *AhDFR* was upregulated, causing substantial substrate shifts to the flavonol pathway in Z1. As a result of the high substrate accumulation of *AhF3H*, Z1 could accumulate anthocyanin but the combined higher expression of both *AhDFR* and *AhF3H* allowed more anthocyanin to be accumulated, with these results being consistent with those for total anthocyanin content and metabolome analysis.

Cui et al. (2016) reported that the upregulation of *LDOX* could increase the anthocyanin content in purple podzolic lentils. In this study, *AhLDOX* was highly expressed in G2 and Z1, and it further catalyzed the formation of anthocyanin from the substrates in G2 and Z1, resulting in the deepening of testa color. This indicated that *AhLODX* was a key gene determining the anthocyanin content as indicated by the phenotypic results. *ANR* and *LAR* are also key genes for the formation of proanthocyanin as they negatively regulate proanthocyanin synthesis (Albert et al., 1997). Additionally, the high expression of *AhLAR* in Z2 increased its proanthocyanin content as reflected in the results of metabolome analysis. In this study, *AhANR* was downregulated in G1_vs_Z1 as well as in G2_vs_Z2, resulting in a decrease of proanthocyanin content in Z1. Combined with the metabolome analysis where both proanthocyanin B2 and B3 were downregulated and proanthocyanin A1 was upregulated in G2_vs_Z2, it can be suggested that *AhANR* could be a key gene that regulates proanthocyanin B2 and B3. Based on the metabolic data, the purple testa at 45 DAF, identified as purple-red by the phenotype identification, could be ascribed to the regulation of proanthocyanin. Proanthocyanin is reddish brown in color, but it can darken the testa color (Li, 2014). On the other hand, the low abundance of blue delphinidin-like substances in Z2 further deepens the red color of testa but these differences need to be further investigated.

**FIGURE 7:**
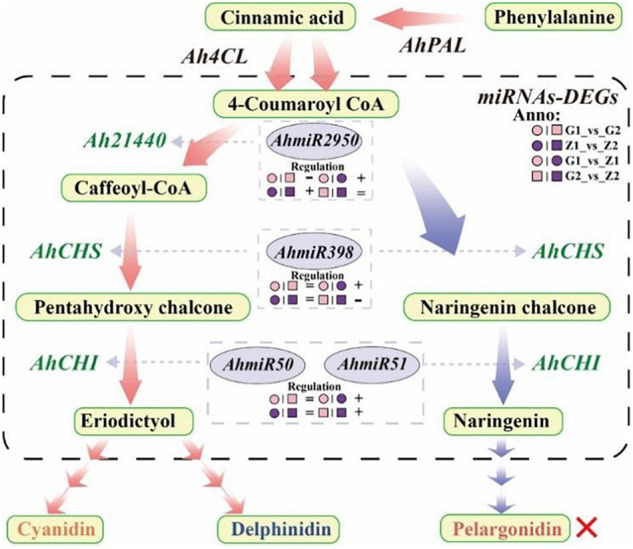
Flow-process diagram of anthocyanin biosynthesis and miRNA interaction with target genes. The picture shows that rectangles with light yellow shading mean metabolites, light red arrows between metabolites mean preferred pathways, and light blue arrows indicate non-preferred pathways yet. The dashed rectangle indicates miRNAs-DEGs interaction. The green labels beside the arrows mean DEGs, the miRNAs corresponding to DEGs are marked in a gray ellipse, and both of them are connected by light gray arrows, and the regulation is indicated below the miRNAs. “°” with pink and purple means 30DAF of G110 and Z18-40, respectively. “□” with pink and purple means 45DAF of G110 and Z18-40, respectively. “+,” “-,” and “=” indicate that there is a positive, negative and none regulatory relationship between DEGs and miRNAs in the comparison on the left. The red cross indicates that there are no regulation expression in the metabolites or genes.

In this study, *Ah21440* was a functional novel gene, which catalyzed the transfer of hydroxycinnamoyl transferase (HCT) to form anthocyanin (Sun et al., 2018). Transcriptome and qRT-PCR results showed that *Ah21440* was upregulated in G1_vs_G2 and Z1_vs_Z2, whilst being downregulated in G1_vs_Z1 and G2_vs_Z2. These results indicated that *Ah21440* continued to be highly expressed as the reproductive period progressed, and that both pink and purple testa favored the formation of caffeoyl coenzyme A before the formation of delphinium and cyanidin. However, no pelargonidin was identified and this was consistent with the metabolome data. This observation may be similar to the substrate specificity of *DFR* and the pathway preference of *HCT* (Yoshida et al., 2010), which needs to be further investigated (Figure 7).

### Interaction Between miRNA and Target Genes

Anthocyanin contribute to resistance to abiotic stress (Valerio et al., 2021). In particular, *ACO3* and *ADT* are involved in responses to various abiotic stresses, while *NPR1* can promote the binding of *TAG* and *PR1*, thus improving plant stress resistance (Johnson et al., 2003). In this study, all nine miRNAs interacted with the target genes to varying degrees and therefore, the results indicated that miRNAs were capable of regulating anthocyanin-based resistance to abiotic stress. The miRNAs involved in the regulation of purple testa were significantly richer than those of pink testa. It was suggested that a potentially positive correlation between testa color and resistance to abiotic stresses. Furthermore, cytochrome P450 could catalyze the synthesis and metabolic reactions of terpenoids, fatty acids, flavonoids and isoflavones (Schuler and Werck-Reichhart, 2003). Additionally, 34 *AhCYP* genes were identified as being involved for catalyzing the hydroxylase and synthase of anthocyanin biosynthesis, and their corresponding nine miRNAs were involved in the regulation of the process in varying degrees. It is likely that miRNAs could regulate the enzymatic functions of anthocyanin biosynthesis. In fact, *AhmiR2950*, *AhmiR5658*, *AhmiR4993*, and *AhmiR5227* regulate both the anti-biotic stress function and the biosynthetic enzyme function of anthocyanin, indicating that miRNAs also have a multi-causal function.

It was shown that *miR398* was involved in regulation through *AP2* and *SPL3* target genes (Li et al., 2013), while miR398_x was involved in the regulation of *F3*′*H* target genes (Hu et al., 2021b). In this study, *AhmiR2950* regulated *Ah21440*, *AhmiR398* regulated *AhCHS* while the *AhmiR50* and *AhmiR51* regulated *AhCHI*. Combined with the transcriptomes data, it was found that *AhmiR2950* is negatively regulated in peanuts with low anthocyanin content, and positively regulated in peanuts with high anthocyanin content and the regulation period tends to be early in development. Additionally, *AhmiR398* was positively regulated at 30 DAF, but negatively regulated at 45 DAF. Both *AhmiR50* and *AhmiR51* were positively regulated on 30 DAF and 45 DAF. These results indicated that the regulatory pattern of miRNAs may vary depending on the variety and the progression of the reproductive period (Figure 7).

## Data Availability Statement

The datasets presented in this study can be found in online repositories. The names of the repository/repositories and accession number(s) can be found below: https://www.ncbi.nlm.nih.gov/, PRJNA773958.

## Author Contributions

GM and XY developed the concept, planned, coordinated, and executed the research. JL wrote the manuscript. JL and YM performed most of the experiments and analyzed the data. YZ, BL, CW, MZ, and LZ prepared the Figures 1–7. GM, XY, and MH revised the manuscript. All authors read and approved the final manuscript.

## Conflict of Interest

The authors declare that the research was conducted in the absence of any commercial or financial relationships that could be construed as a potential conflict of interest.

## Publisher’s Note

All claims expressed in this article are solely those of the authors and do not necessarily represent those of their affiliated organizations, or those of the publisher, the editors and the reviewers. Any product that may be evaluated in this article, or claim that may be made by its manufacturer, is not guaranteed or endorsed by the publisher.

## Footnotes

1
http://www.illumina.com

2
https://v1.legumefederation.org/data/public

3
https://v1.legumefederation.org/data/public/Arachis_hypogaea/Tifrunner.gnm1.ann1.CCJH/

4
http://www.hmdb.ca/

5
http://appliedbioinformatics.wur.nl

6
http://metlin.scripps.edu/index.php

7
https://cipotato.org/genebankcip/

8
http://mirbase.org/

9
http://sourceforge.net/projects/mireap/
