# Corrigendum: Multi-Omics and miRNA Interaction Joint Analysis Highlight New Insights Into Anthocyanin Biosynthesis in Peanuts (*Arachis hypogaea* L.)

**DOI:** 10.3389/fpls.2022.929085

**Published:** 2022-05-16

**Authors:** Jiawei Li, Yucong Ma, Mengdie Hu, Yulu Zhao, Bin Liu, Chunmei Wang, Min Zhang, Liping Zhang, Xinlei Yang, Guojun Mu

**Affiliations:** State Key Laboratory of North China Crop Improvement and Regulation, North China Key Laboratory for Crop Germplasm Resources of Education Ministry, Laboratory of Hebei Provincial Crop Germplasm Resources, Hebei Agricultural University, Baoding, China

**Keywords:** peanut, anthocyanin, testa, multi-omics joint analysis, miRNA interaction, qRT-PCR

In the original article, there were two mistakes in Figure 7 and Supplementary Figure 1 as published. The author mistakenly wrote “AhmiRNA398” as “AhmiRNA50” in Figure 7 and the paternal parent “Zizhenzhu” had been wrongly written as “Z18-40” in Supplementary Figure 1, which caused a discrepancy with the original text and affected readers' understanding. The corrected Figure 7 and Supplementary Figure 1 appears below.

**Figure 7:**
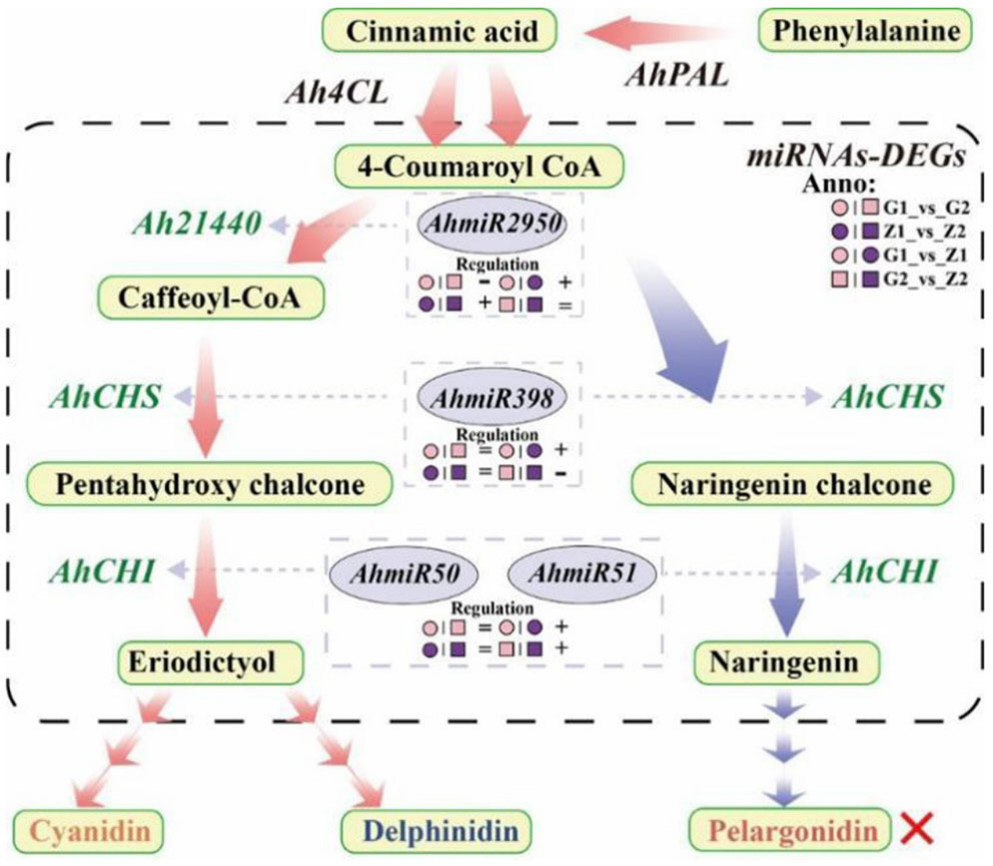
Flow-process diagram of anthocyanin biosynthesis and miRNA interaction with target genes. The picture shows that rectangles with light yellow shading mean metabolites, light red arrows between metabolites mean preferred pathways, and light blue arrows indicate non-preferred pathways yet. The dashed rectangle indicates miRNAs-DEGs interaction. The green labels beside the arrows mean DEGs, the miRNAs corresponding to DEGs are marked in a gray ellipse, and both of them are connected by light gray arrows, and the regulation is indicated below the miRNAs. “°” with pink and purple means 30DAF of G110 and Z18-40, respectively. “□” with pink and purple means 45DAF of G110 and Z18-40, respectively. “+,” “-,” and “=” indicate that there is a positive, negative and none regulatory relationship between DEGs and miRNAs in the comparison on the left. The red cross indicates that there are no regulation expression in the metabolites or genes.

**Supplementary Figure 1:**
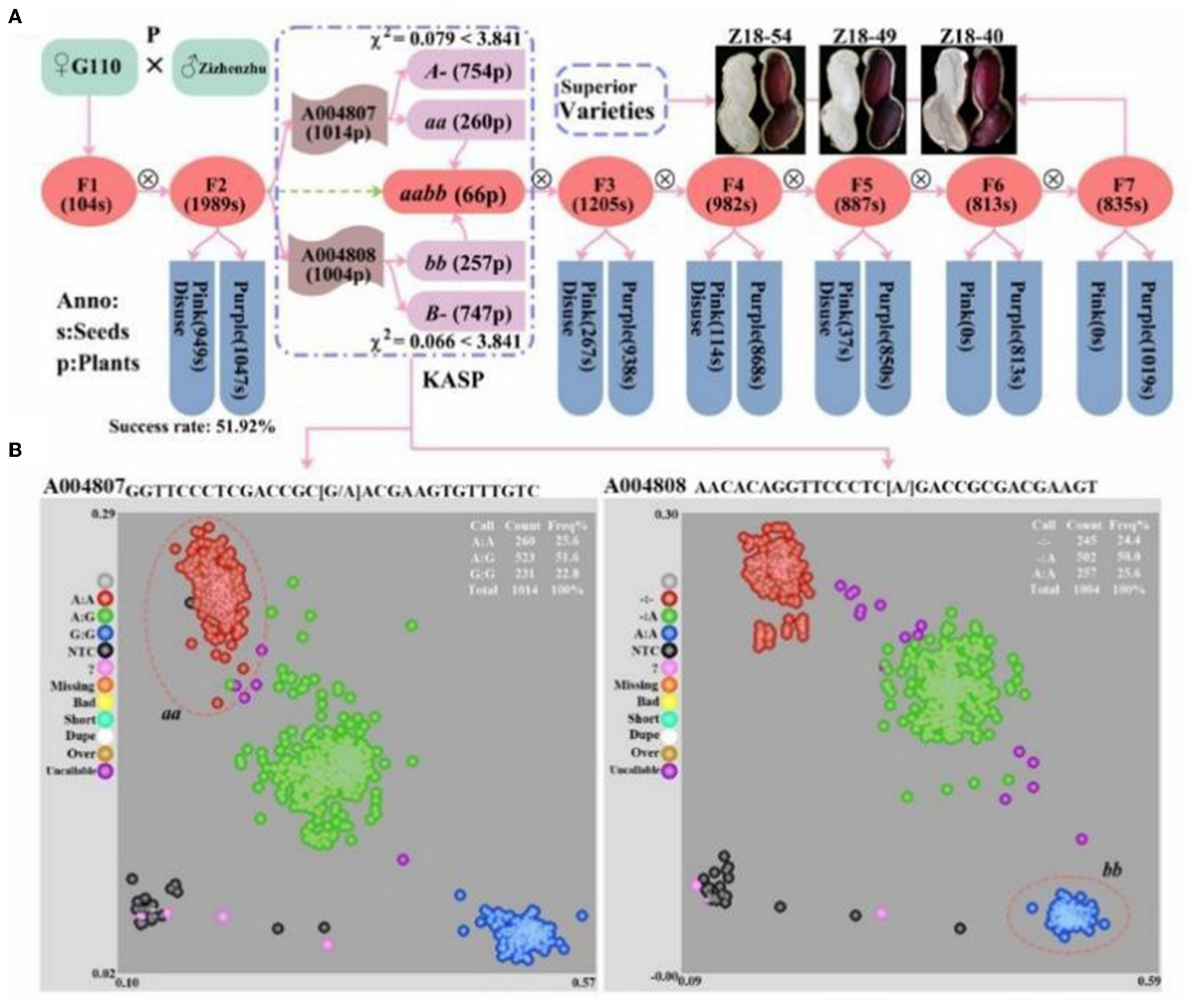


The authors apologize for this error and state that this does not change the scientific conclusions of the article in any way. The original article has been updated.

## Publisher's Note

All claims expressed in this article are solely those of the authors and do not necessarily represent those of their affiliated organizations, or those of the publisher, the editors and the reviewers. Any product that may be evaluated in this article, or claim that may be made by its manufacturer, is not guaranteed or endorsed by the publisher.

