# Supplementary figures and images for: Multi-Omics and miRNA Interaction Joint Analysis Highlight New Insights Into Anthocyanin Biosynthesis in Peanuts (Arachis hypogaea L.)
Source: Front Plant Sci. 2022 Feb 16;13:818345. doi: 10.3389/fpls.2022.818345 (PMC8888885; doi:10.3389/fpls.2022.818345)

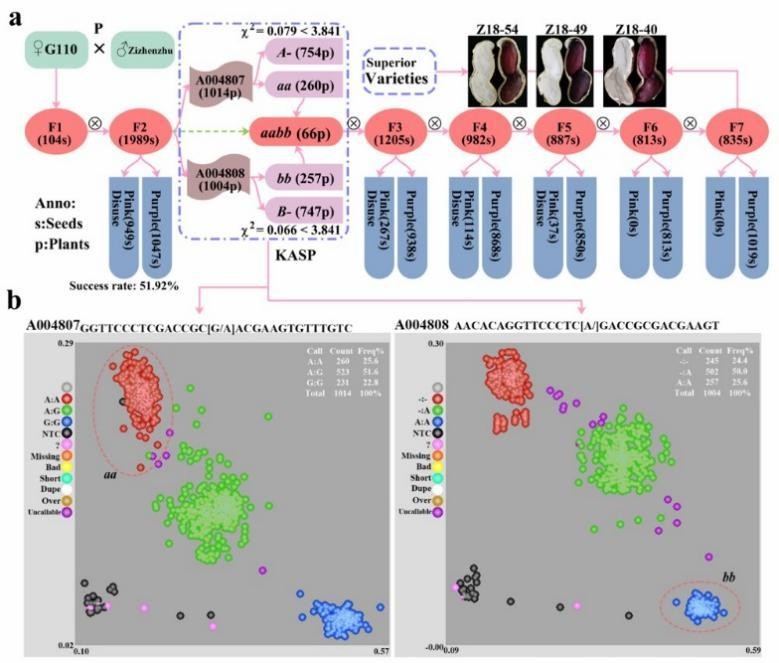

Supplement: Supplementary file 11 [file Image_1.JPEG]

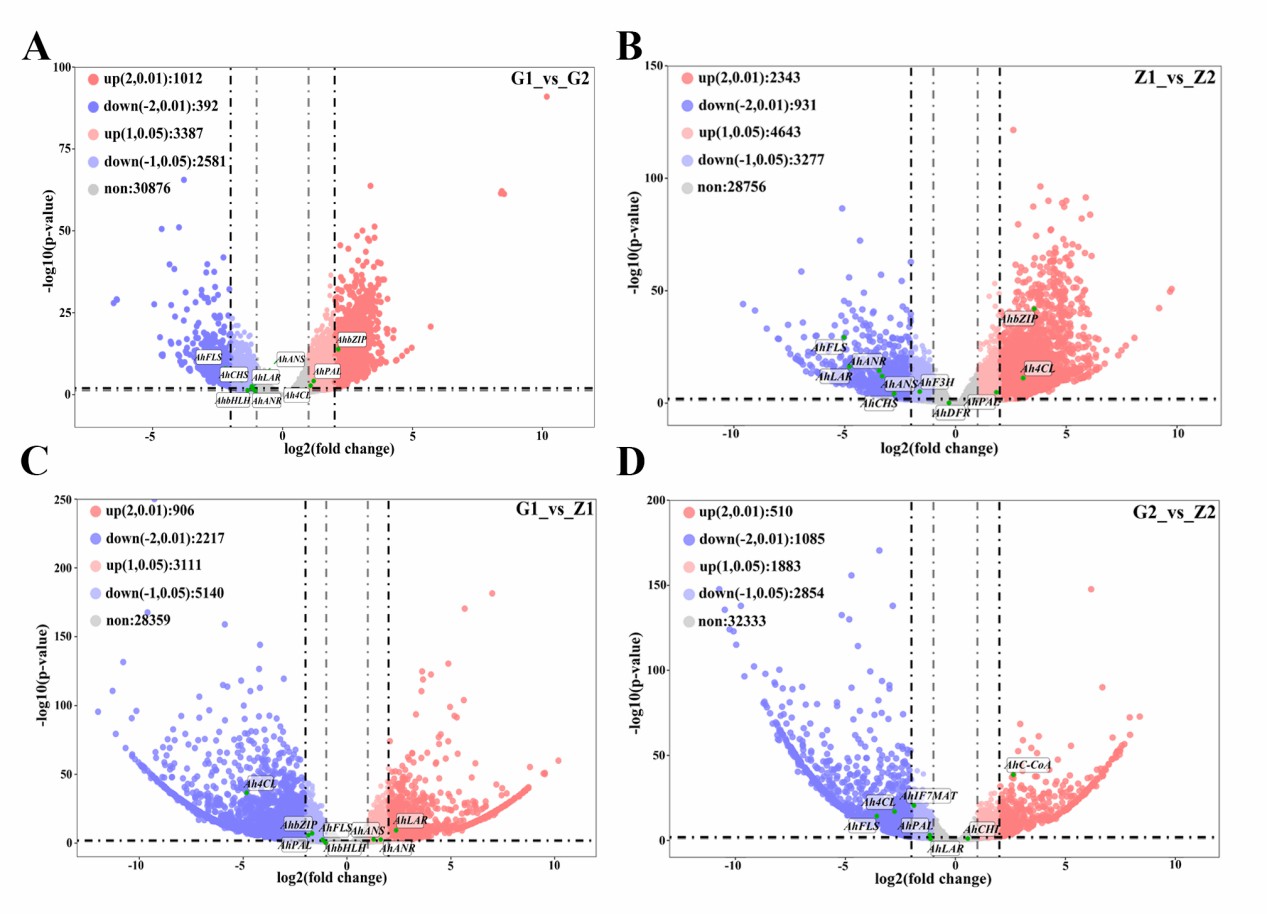

Supplement: Supplementary file 12 [file Image_2.JPEG]

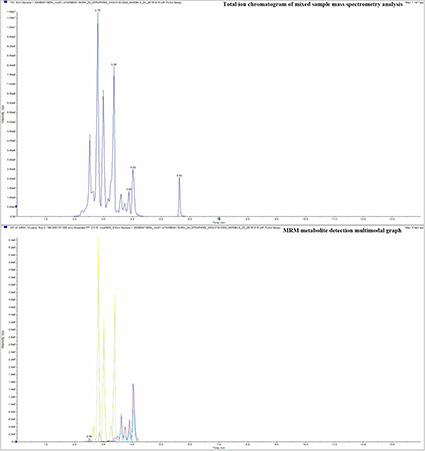

Supplement: Supplementary file 13 [file Image_3.JPEG]
